# *import_pentacam*: Open-Source OCR Recovery of Corneal Tomography Measurements From Pentacam DICOM, PDF, and Image Archives

**DOI:** 10.1167/tvst.15.10.46

**Published:** 2026-10-27

**Authors:** Katerina Kiroshka, Tobias Elze

**Affiliations:** 1 Lumael Eye Medical Institute Ltd., Ashkelon, Israel; 2 Schepens Eye Research Institute of Mass. Eye and Ear, Department of Ophthalmology, Harvard Medical School, Boston, MA, USA

**Keywords:** keratoconus, corneal ectasia, Pentacam, Scheimpflug tomography, DICOM, optical character recognition, longitudinal dataset, open-source, research infrastructure

## Abstract

**Purpose:**

Pentacam corneal tomography data accumulate in clinical Digital Imaging and Communications in Medicine (DICOM) archives as embedded portable document format (PDF) printouts and proprietary binary readouts rather than structured records; the device’s comma-separated values (CSV) export is impractical for retrospective multisite use. We describe an open-source pipeline that recovers structured Pentacam measurements from archives.

**Methods:**

*import_pentacam* is an open-source Rust tool that extracts up to 66 numerical fields and embedded corneal-map images per examination from DICOM, PDF, or image inputs, with single-file, file-list, and recursive picture archiving and communication system (PACS) directory modes supporting continuous incremental updates. Cross-firmware layout variation is normalized by affine transforms onto printout templates. The pipeline was developed on 300 examinations across three printout types and evaluated on a 200-examination holdout; Belin/Ambrósio Ectasia was added as a new printout type without retraining and tested on 333 examinations. Ground truth was derived from automated annotators with manual review of disagreements.

**Results:**

Field-level accuracy was 99.46% on training, 98.70% on the holdout, and 99.03% on the Belin/Ambrósio corpus. Agreement with paired DICOM Structured Reports was 100% on 518 fields. Institutional deployment recovered 126,184 eye visits from 29,550 patients across an 18-year archive.

**Conclusions:**

Automated recovery of structured Pentacam data from clinical DICOM archives is feasible at large multisite institutional scale. The software supports continuous incremental updates and is deployable at other Pentacam-equipped institutions for longitudinal ectasia research.

**Translational Relevance:**

Routinely collected institutional Pentacam examinations are a substantial resource for longitudinal corneal tomography research. This pipeline converts that resource into structured data without disrupting clinical workflows, enabling continuously updated retrospective cohorts and federated extraction across institutions.

## Introduction

Keratoconus and corneal ectatic disease are a growing clinical burden. Recent meta-analyses place global keratoconus prevalence between 138 and 289 per 100,000,^1^^,^^2^ and the Corneal Ectasia Preferred Practice Pattern^3^ summarizes current evidence and recommended care for keratoconus and related ectatic disease. Critical clinical questions—which eyes will progress, at what rate, and which patients benefit most from preventive crosslinking before irreversible structural change—require longitudinal corneal tomographic data at a scale that existing research datasets do not provide.

Pentacam rotating Scheimpflug tomography (Oculus GmbH, Wetzlar, Germany) has become the clinical standard for corneal ectasia evaluation and refractive surgery screening.^4^^,^^5^ Each examination produces a dense anterior-segment phenotype, including anterior and posterior corneal curvature, pachymetric maps, elevation deviation from reference surfaces, anterior chamber geometry, and composite ectasia risk scores, most notably the Belin/Ambrósio Enhanced Ectasia Display (BAD-D)^6^ and the ABCD grading system.^7^^,^^8^ No other widely deployed clinical instrument generates an equivalent density of corneal tomographic parameters in a single acquisition.

Despite this clinical richness, secondary research use of Pentacam data is limited by a structural data-access problem. In most clinical institutions, Pentacam outputs reach the picture archiving and communication system (PACS) as embedded PDF printouts within Digital Imaging and Communications in Medicine (DICOM) objects or as proprietary binary readouts, rather than as natively structured database records; electronic health records typically capture only a handful of fields (commonly K1, K2, and thinnest pachymetry). The Pentacam console itself does expose a comma-separated values (CSV) export, but it is operator-driven and requires *physical access* to the device at the time of export. At single-device clinics, this can be operationalized; in our own institutional setting—a tertiary referral center operating more than 10 Pentacam devices across multiple satellite clinical sites, with examinations accumulating continuously since 2008—and in any similar multisite deployment in which research access to clinical instruments is restricted, manual device-side export is neither practical nor retrospective. The PACS archive is therefore the only unified, automatically populated layer through which Pentacam measurements from across an institution can be reached, and the structured-export problem is, in practice, a PACS-extraction problem.

The scale consequence of this access barrier is visible throughout the literature. The largest multicenter Pentacam ectasia validation cohort involved 3886 eyes from 26 international sites;^6^ the largest published single-center ABCD baseline cohort comprised 1917 corneas;^8^ the REDCAKE multicenter project enrolled 1155 eyes from 906 patients with keratoconus across eight contributing centers.^9^ Even the largest natural history meta-analyses pool data across many small independent cohorts,^10^ with no single-source longitudinal dataset of substantial scale. The gap between available data and what robust modeling requires is infrastructural rather than biological.

Automated extraction of clinical report data has been demonstrated in other domains.^11^^,^^12^ The precedent directly relevant to ophthalmology is the automated extraction of numerical data from Humphrey Visual Field printouts,^13^ which showed that report-embedded ophthalmic measurements can be recovered at high accuracy from a single printout type. To our knowledge, no equivalent pipeline has been described for Pentacam, which presents distinct challenges: multiple printout types whose layouts vary with firmware version, numerically dense tabular content, an LCD-style hollow-stroked digit font that is rasterized into the report rather than embedded as text, and variable archiving quality across PACS configurations (including low-resolution JPEG exports and pre-2010 layouts).

We describe the development, validation, and institutional-scale deployment of *import_pentacam*, an open-source pipeline that recovers structured Pentacam data from clinical DICOM archives. We report field-level accuracy across three validation corpora, including an internal holdout, independent verification against machine-precision DICOM SR values, and the result of a continuously updated deployment at our institution that has recovered measurements from approximately 30,000 patients across an 18-year archive. (DICOM Structured Reports, abbreviated SR throughout, are the standardized numerical-measurement section of a modern Pentacam DICOM object; we use the standard abbreviation from firmware version 1.30 onward.)

## Methods

### Software Overview

The software *import_pentacam* is released under the AGPL-3.0 open-source license at https://github.com/tobiaselze/import_pentacam. It is implemented in Rust as a single statically linked binary; runs on Linux, macOS, and Windows without runtime installation of any external dependency; and uses an NVIDIA GPU when one is available (with automatic fallback to the CPU otherwise). From each examination, it extracts up to 66 numerical fields covering 61 distinct clinical parameters, drawn from three complementary on-disk data sources: the DICOM Structured Report (SR), the Oculus proprietary binary readout (an internal file with extension .SPR, referred to throughout simply as the SPR), and optical character recognition (OCR) of the embedded PDF printouts. Values from the multiple sources are merged per eye visit by a fixed priority rule (below). Institutional deployment of the software at one tertiary referral center is described in the Results as a usage example. The overall architecture of the software is shown in Figure 1.

**Figure 1.**
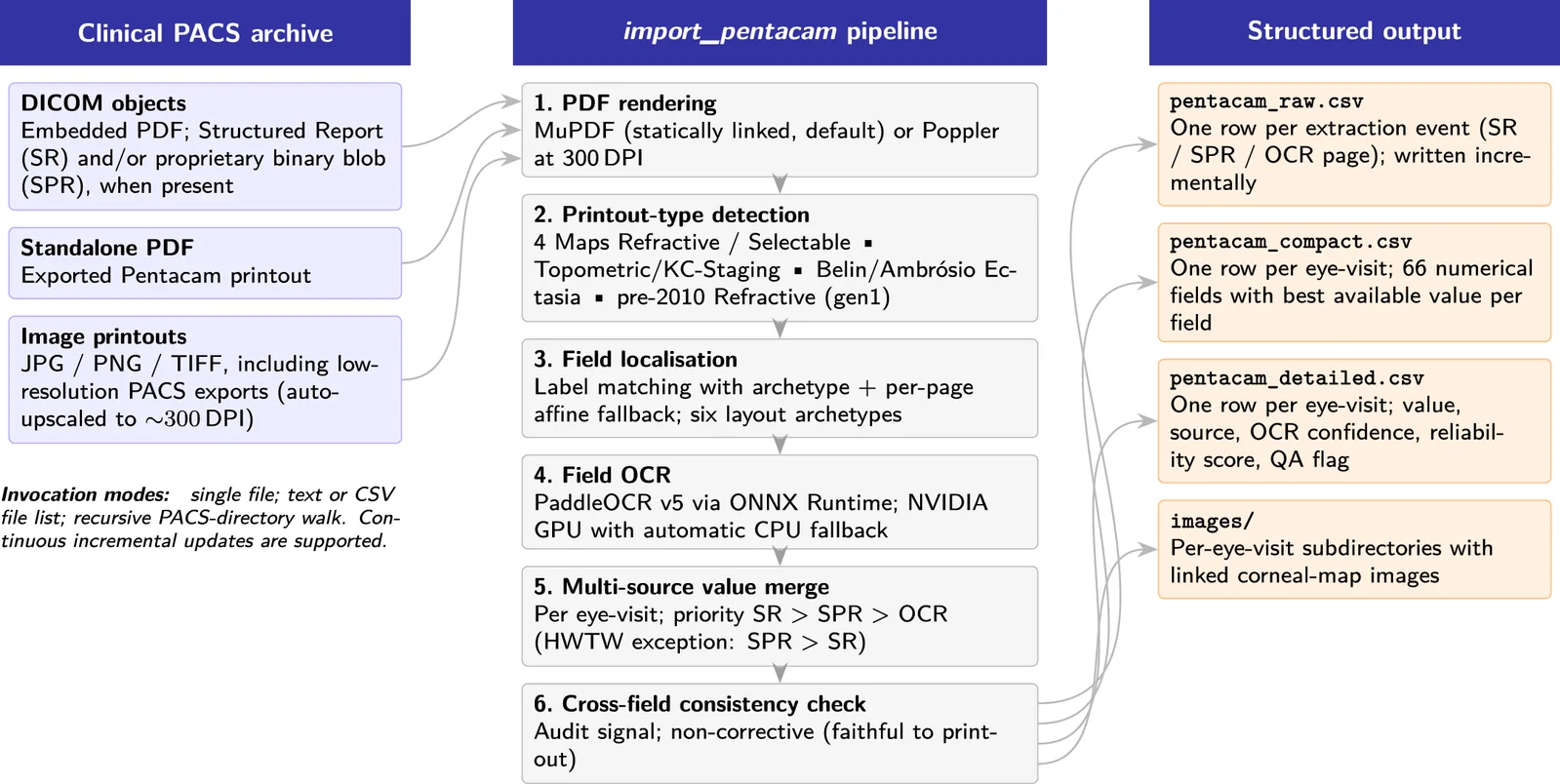
Architecture of the *import_pentacam* software. Three input formats from the institutional PACS—DICOM objects, standalone PDF files, and image-format printouts (JPG, PNG, or TIFF, including low-resolution PACS exports that are automatically upscaled before OCR)—pass through six pipeline stages: PDF rendering, printout-type detection, field localization by label matching with archetype-plus-affine fallback, field-level OCR, multisource value merge, and cross-field consistency check. Within DICOM input, the proprietary SPR binary blob and the DICOM Structured Report (SR)—when either is present—provide partial machine-precision measurements that are merged with OCR readings per eye visit under the priority SR > SPR > OCR, with one field-specific exception for the white-to-white cornea-diameter field HWTW, for which the SPR readout is preferred over SR. Output is three CSV files differing in per-row aggregation level, plus per-eye-visit subdirectories of linked corneal-map images. The italicized note below the input column indicates that the software can be invoked on a single file, a text or CSV file list, or a recursive walk of a PACS-directory tree and supports continuous incremental updates. DPI, dots per inch; GPU/CPU, graphics/central processing unit; ONNX, Open Neural Network Exchange; QA, quality assurance.

### Supported Inputs and Printout Types

The software accepts three input formats: (1) DICOM objects (containing embedded PDF, SPR blob, and/or SR), (2) standalone PDF files, and (3) image printouts (JPEG, PNG, TIFF), including the low-resolution JPEG exports characteristic of legacy PACS archiving. Input format is auto-detected by magic bytes, allowing uniform processing of heterogeneous archives. Files with nonstandard filename extensions (e.g., the .JPG_1 suffix produced by some PACS servers) are identified by content rather than name. Low-resolution image inputs are automatically upscaled to approximately 300 DPI using Lanczos3 interpolation before OCR.

Five Pentacam printout types are supported: the *4 Maps Refractive* and *4 Maps Selectable* displays (anterior and posterior keratometry, pachymetry, anterior chamber, corneal volume), the *Topometric/KC-Staging* display (keratoconus indices, Kmax, ARTmax, and the True Net Power section), the *Belin/Ambrósio Enhanced Ectasia Display* (BAD-D composite score and sub scores *D*_*f*_, *D*_*b*_, *D*_*p*_, *D*_*t*_, *D*_*a*_, elevation and pachymetric progression indices, ARTmax), and a pre-2010 generation-1 Refractive printout (*Refractive gen1*). Up to 66 numerical fields are extracted per examination—40 from the 4 Maps Refractive/Selectable and Topometric set, 6 from the True Net Power block on Topometric pages, and 20 from the Belin/Ambrósio display—covering 61 distinct clinical parameters; the remaining 5 fields are independent Belin-display readings of keratometry parameters (K1, K2, KMax, Axis, Qval) that also appear on the 4 Maps printouts and are retained as cross-page consistency checks.

### Validation Corpora

We assembled three disjoint Pentacam DICOM corpora drawn from the institutional archive at Massachusetts Eye and Ear, Boston, Massachusetts (Mass General Brigham IRB protocol 2020P001232; consent waived). The *training corpus* comprises 300 examinations spanning the three primary 4 Maps/Topometric printout types, covering multiple Pentacam firmware versions and several physical devices, and was used during pipeline development. The *holdout corpus* comprises 200 examinations drawn from a separate date range and PACS configuration, with no overlap with training and no access during pipeline development. The *Belin corpus* comprises 333 examinations used for the Belin/Ambrósio extension, which was added only after the core pipeline had been frozen. Adding it required a new layout archetype and a new field-label list; the existing crop and OCR machinery were reused unchanged. The 333 examinations were processed in two disjoint batches, each stratified equally across the same 9 archive months (September 2021–February 2026): an initial 135-examination batch, on which the single Belin-specific implementation choice was finalized (a fixed-size field crop replacing the size-adaptive crop used for the denser primary layouts), and a subsequent, held-out 198-examination batch used only for evaluation once the extension was frozen. All three corpora were sampled to over represent rare layout variants (low-resolution PACS exports, pre-2010 firmware printouts) so that pipeline robustness, and not only aggregate accuracy, could be evaluated. Each corpus is a random subset of the institutional archive; the institutional-scale deployment reported in the Results is therefore an application of the software on the same archive from which the validation corpora were drawn, on the orders-of-magnitude-larger remainder.

### Multisource Value Priority Hierarchy

A single examination can yield several data sources. DICOM objects with firmware ⩾1.30 carry an SR containing 15 of the 66 clinical fields at machine precision in a coded ContentSequence: HWTW, anterior chamber depth (AC_depth), pupil diameter, Kmax (encoded as the minimum axial radius of curvature and converted via *K* = 337.5/*R*), the six Belin/Ambrósio BAD-D scores (*D*_*f*_, *D*_*b*_, *D*_*p*_, *D*_*t*_, *D*_*a*_, *D*_final_), the three progression indices (PI_min_, PI_max_, PI_avg_), the ARTmax pachymetric progression index, and the thinnest pachymetry. DICOM objects with firmware ⩽1.28 do not carry an SR; they instead include the SPR, a proprietary 38,400-byte Oculus binary blob stored in the private DICOM tag (0029,1010). A separate larger private tag (0029,1020; approximately 5.9 MB) holds raw Scheimpflug image data in a proprietary, undocumented format and is not processed by the software. A subset of firmware ⩾1.31 DICOMs—which we refer to here as “Rosetta” files because they expose the same measurements in two independent encodings, an SR and an SPR side-by-side—retain both. In our institutional archive, the SPR was present and parsed in 93,565 of 421,423 raw extraction events; of these, 1389 were Rosetta firmware ≥1.31 cases that also carried an SR.

The SPR likely encodes the full set of clinical measurements made by the device; the format is, however, proprietary, and we have not undertaken a systematic reverse-engineering of it. We extract only those values whose location and binary encoding in the SPR are identifiable directly from the structure of the file. Two fields meet this criterion. The first is the cornea diameter (the HWTW field), stored as an unsigned 16-bit integer at fixed offset 0x05BA (in units of 0.01 mm). The second is the anterior chamber depth (AC_depth), which can be read exactly as the difference of two double-precision floats stored at fixed offsets in the blob (in our notation, a1[0][1] - a1[0][4] in the structure’s first array; a single-column shift applies on one binary-format variant). The SR, in contrast, is a standardized DICOM object and is parsed for all clinical fields it exposes. OCR of the embedded PDF is the universally available source and is run on every printout.

Each source is recorded individually as a row in the raw output (one row per extraction event); the multiple sources are then merged per eye visit with priority **SR > SPR > OCR**. One field-specific exception is applied: HWTW prefers SPR over SR, because the SPR encodes the cornea-diameter measurement as an exact 16-bit integer, and the SR value is in this case a re-encoding of the same underlying datum. When SR and OCR disagree beyond a field-specific tolerance, a structured SR_OCR_MISMATCH flag is set in the per-eye detailed output to support downstream quality review.

### Page Rendering

Pentacam PDF printouts do not represent their measurement values as PDF text or vector content: the entire data region of every printout type we have inspected, including all numerical values, is rasterized into the PDF as bitmap content at PDF-generation time. OCR of the rasterized page is therefore the only available readout mechanism, and the choice of OCR is dictated by the source format rather than being a workaround for it. We render the embedded PDF at 300 DPI before OCR. The software uses MuPDF^14^ as its PDF renderer; the MuPDF library is statically linked into the binary, so no separate PDF runtime needs to be installed on the user’s system. A Poppler^15^ renderer is selectable via a build flag.

### Layout Normalization: Label Matching With Archetype Fallback

The Pentacam report layout is organized into a small number of field groups (see Fig. 2): a left-hand column containing anterior and posterior keratometry, pachymetry with field coordinates, and corneal and anterior chamber volumes, alongside a right-hand grid of four corneal-map images. The layout varies across firmware revisions: column spacing, font metrics, and the position of individual labels shift between firmware eras. Two strategies for locating each numerical value on the page are conceivable: (a) match the literal label string (“K1:,” “Astig.:,” etc.) on the page and read the value from the adjacent crop, or (b) maintain a hard-coded list of expected pixel locations per printout type (an *archetype*) and read the value from the archetype location.

**Figure 2.**
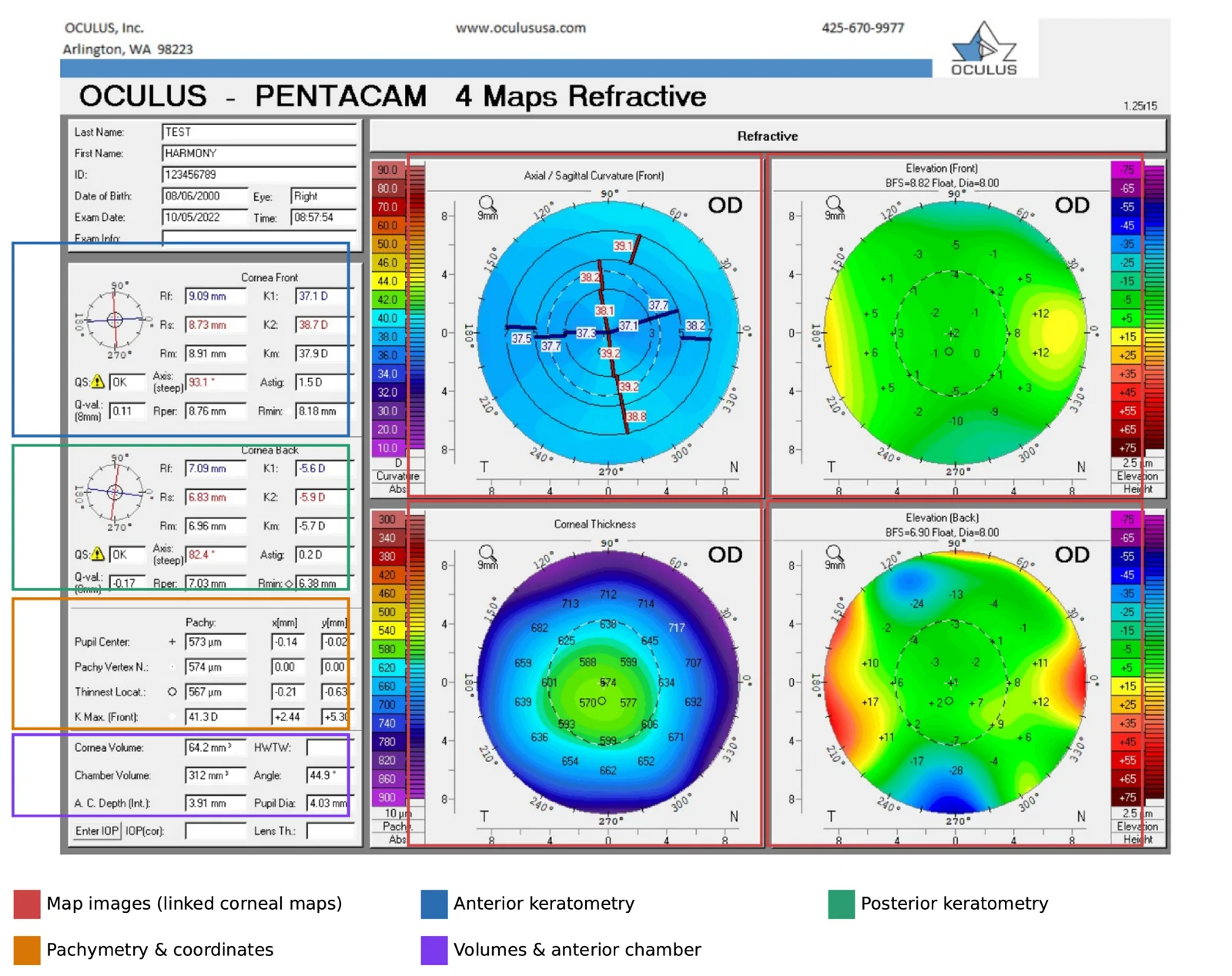
Annotated 4 Maps Refractive Pentacam printout illustrating the field-group layout used by *import_pentacam*. The colored overlays mark the regions from which the software reads the numerical parameters of an examination: anterior keratometry (*blue*), posterior keratometry (*green*), pachymetry and field coordinates (*orange*), and corneal/anterior chamber volumes (*purple*). The red outlines mark the four corneal-map images (axial/sagittal curvature, anterior elevation, corneal thickness, posterior elevation) that are extracted as PNG files alongside the numerical CSV output. Within each numerical region, individual field values are localized either by label matching on the page or, when that fails, by a per-page affine transform from the archetype’s reference field positions. The image shown is a rendering of the embedded PDF from a deidentified Pentacam DICOM file (synthetic patient identifiers).

We use both, with label matching as the default. Each page is OCR’d once to obtain a list of all detected text items with their pixel positions; the software then searches this list for each known field label and reads the value adjacent to the matched label. When a label cannot be matched on the page—because the OCR detector missed it, or because the layout has shifted such that the label string is rendered differently—the software falls back to the archetype.

The archetypes currently shipped cover the 4 Maps Refractive/Selectable and Topometric printouts under two firmware-era variants (Group A, firmware ⩽1.28; Group B, firmware ⩾1.30, separated by the rebranding-era column geometry change between the 1.28 and 1.30 firmware releases), a low-resolution PACS-export variant (902–904 px page height, with nonuniform vertical stretch), the pre-2010 generation-1 Refractive layout, and two variants for the Belin/Ambrósio Ectasia display. For each printout type, the archetype is a hard-coded list of expected (field, pixel-position) pairs measured on a representative reference page; the per-page affine transform mapping the archetype to the live page is fitted, with iterative outlier rejection, from the labels that *were* matched on the page (vertical scale α, vertical offset β, plus horizontal equivalents). The residual standard deviation of the fit is recorded as a per-page quality metric: a high residual means that no shipped archetype is consistent with the page layout, and the examination is flagged for review.

The archetypes are clearly delimited in the source code and are straightforward to extend. Adding support for a new printout type, or for a new layout variant of an existing printout introduced by a future firmware release, consists of measuring the field positions on a representative reference page and adding the new archetype entry; this is the principal extensibility mechanism of the software. Across the 200-file internal holdout corpus, every page was matched to one of the shipped archetypes within tolerance, providing initial evidence that archetype coverage is complete for the supported printout types in the current archive.

### Crop-Based Reading and the OCR Engine

Once a field’s pixel position on the page has been determined (by label matching or by the archetype fallback), the software extracts a tight crop around that position (typically ±70 px horizontally and ±20 px vertically), normalizes colored cell backgrounds and the LCD-style outlined digit strokes to standard black-on-white, fills hollow digit interiors morphologically, upscales 3×, and reads the value with PaddleOCR v5^16^ via ONNX Runtime.^17^ PaddleOCR comprises a DBNet-style text detector, followed by an SVTR text recognizer over a closed character set; because recognition is cast as sequence classification over a fixed vocabulary, PaddleOCR cannot insert characters that are not supported by the image evidence, and a per-token recognition confidence is available for every read. The software automatically uses an NVIDIA GPU via ONNX Runtime’s CUDA execution provider when one is available and silently falls back to CPU otherwise; a command-line flag is provided to force CPU execution on machines that do have a GPU. Per-page OCR runtime is approximately 5 seconds on a current desktop NVIDIA GPU and is several tens of seconds per page on a CPU, dominated by the SVTR recognition step.

The released software uses PaddleOCR alone as its OCR engine. No vision–language model (VLM) is invoked at inference time. A VLM is used as an independent second reader during ground-truth generation only; see below.

### Cross-Field Consistency Check

After extraction, fields whose values are mathematically related are checked for consistency: mean keratometry as the average of flat and steep meridians, the radius-to-power conversion *K* = 337.5/*R*, paired anterior/posterior radii, and similar identities. The software’s design goal is to reproduce the printout faithfully, not to “correct” it: in several examinations, the printout itself displays internally inconsistent values, most commonly when the device’s underlying measurement failed and the device-side computation overflowed or emitted a sentinel value; in those cases, the software records the displayed value as it appears, even where it violates the mathematical relationship. The cross-field consistency check is therefore informational rather than corrective: every violating examination is logged with a structured warning, which supports downstream sensitivity analyses and which we use as an automated audit signal in the validation work described below.

### Incremental and Restart-Safe Operation

The software supports four input modes: a single file, a text file list, a CSV file list with optional metadata columns, and a recursive PACS directory walk. All modes are restart-safe: a per-folder or per-file log is updated atomically after the corresponding measurements are flushed to the output CSV, so interrupted runs resume without reprocessing completed work. This design supports a continuous *incremental* deployment, in which a periodically refreshed file list of newly archived examinations is fed to the software; only new files are processed, and the cumulative output CSVs grow monotonically. At our institution the software is operated as a weekly automated update, providing a continuously maintained structured-data resource without disrupting clinical workflows.

### Output Format

Each run produces three output files per output directory. pentacam_raw.csv contains one row per extraction event (one row per SR object, one per SPR blob, one per OCR’d PDF page), written incrementally as work completes. pentacam_compact.csv contains one row per eye visit, with the best available value per field selected by the multisource priority rule, suitable for direct import into statistical or data-analysis environments. pentacam_detailed.csv contains one row per eye visit with five columns per field: value, source (SR/SPR/OCR), per-token OCR confidence, field-level reliability score (calibrated from validation, embedded in the software), and a QA flag (SR_OCR_MISMATCH, LOW_CONF, REVIEW, OUT_OF_RANGE).

In addition to the structured CSV output, the software extracts the graphical corneal-map images embedded in the rendered PDF printouts: 2 × 2 grids of anterior and posterior elevation maps, sagittal and tangential curvature maps, and pachymetric maps from the 4 Maps printouts, as well as elevation, thickness, and CTSP/PTI chart images from the Belin/Ambrósio display. Each eye visit’s images are written to a per-visit subdirectory whose name is recorded on the same row of the detailed output CSV as the eye visit’s numerical fields, so that researchers can retrieve both structured measurements and the corresponding spatial imaging for any eye visit from a single output file. These map exports are cropped regions of the rendered printout: color-mapped images with their accompanying color-scale bars, at the resolution of the 300 DPI page render. They are directly usable as inputs to image-based deep learning (e.g., convolutional or vision-transformer models operating on the color map) and are spatially consistent across examinations because the crop is defined geometrically from detected layout anchors. They are, however, not raw per-pixel elevation, curvature, or pachymetry value matrices: recovering quantitative micrometer or diopter values at each pixel would require a color-to-value calibration against the printed color-scale bar, or—for the subset of examinations with a DICOM Structured Report—use of the machine-precision scalar values already extracted to the CSV output. We therefore describe the images as analysis-ready for image-based modeling, while noting that pixel-level quantitative use requires this additional calibration step.

### Ground-Truth Generation

Validation of the software requires a reliable per-field ground truth (GT): the correct measurement value on each PDF crop, established independently of any of the readings the software produces. Exhaustive manual annotation of every numerical field on every training examination was infeasible. We generated GT in two stages.

*Stage 1—reliability of the annotation method.* Before using automated annotation at scale, we first established its reliability on a small manually curated subset of 15 examinations (approximately 540 numerical fields). Two independent large-context VLMs, Claude Sonnet and Claude Opus, were each asked to transcribe every field crop using the identical zero-shot prompt: *“Transcribe the exact numeric value printed in this image. Copy exactly what is printed—do not interpret or correct. Reply with ONLY the number, nothing else.”* Three design points for the GT process are worth noting here. First, each VLM saw only a tight single-field crop and never the full page, because generative VLMs can confabulate plausible values from surrounding visual context (other numerical values on the page, patient identifiers) when local image evidence is ambiguous, and a tight crop containing only the digits to be transcribed removes that contextual material. This tight-crop constraint is also a data-minimization safeguard: the cloud-hosted VLMs (Claude Sonnet and Opus) received only a small numerical region containing a single measurement value and no patient-identifying information—no full printout page or header region was transmitted—whereas the released pipeline (PaddleOCR) and the local Qwen reader run entirely on-premises, so production extraction involves no external data transmission. Second, the crop locations for GT were generated using the archetype-plus-affine fallback (without label matching), so that the same pixel region was presented to both VLMs for each field independently of any OCR detection on the page. Third, frontier VLMs are token-intensive and slow on this task—on the order of 1 to 2 minutes of model time per printout page in our setup—and the per-call cost is therefore prohibitive for a full-archive deployment, which motivated the cheaper PaddleOCR–Qwen consensus procedure of Stage 2 for the larger corpora.

The same 15 examinations had also been read independently by a clinically trained human annotator. Where the dual-VLM consensus disagreed with the human reading, we inspected the original crops; in every case, the dual-VLM consensus was correct, and the discrepancies were attributable to human transcription errors (transposition of adjacent rows, fatigue-driven digit substitution). This finding established the dual-VLM consensus as at least as reliable as careful manual reading on this font and motivated its use as the primary GT source before any pipeline accuracy assessment.

*Stage 2—scaled GT generation by PaddleOCR–Qwen consensus.* Scaling Stage 1 to the full training corpus would have required several days of API time. We instead exploited the empirical observation that PaddleOCR and Qwen 2.5-VL-7B,^18^ applied to the same tight field crop, fail in mostly uncorrelated ways. For every crop (again generated from the archetype-plus-affine fallback, without label matching), we ran both engines and partitioned the results into an *agree bucket* (both engines returned identical normalized values; accepted as GT without further review, for the corpora generated by this procedure) and a *disagree bucket* (engines disagreed, or one returned no read; reviewed by a human using a desktop tool that displays the crop alongside both engines’ transcriptions). Across the 300 training examinations, 11,488 numerical fields fell into the agree bucket; the remaining fields were resolved in the disagree bucket and contributed the bulk of the human annotation effort. We emphasize again that the VLM-based stack described in Stages 1 and 2 is used only to generate ground truth for validation; the released software uses PaddleOCR alone. Beyond the 15-examination reliability check, the dual-VLM (Sonnet and Opus) consensus was the reference ground-truth source for the testset3 and new_testset development subcorpora (180 of the 295 training examinations); the PaddleOCR–Qwen consensus of Stage 2 was applied to the sep2021 training subset and to the holdout and Belin corpora. The majority of the training-corpus ground truth was therefore established by models with no relationship to PaddleOCR, so for that portion, the reference is independent of the OCR engine being evaluated.

### Estimating the Residual Error of the Working Ground Truth

The consensus-generated GT described above is an *estimate* of the true measurement value on each printout: it is the value on which two independent readers (PaddleOCR and Qwen 2.5-VL-7B in Stage 2; Claude Sonnet and Claude Opus in Stage 1) agreed, with human review of disagreements, but the true value on the printout was not directly measured by an independent method. To estimate how reliable this working GT is, we applied the cross-field consistency checks described above to the consensus-agreed values themselves. Twenty-three examinations were flagged. Manual inspection of the underlying crops showed that 21 of the 23 reflected printout-level inconsistencies that the consensus had correctly transcribed—the printout itself displayed internally inconsistent values, typically failed measurements rendered as out-of-range sentinels or computational overflow on edge-case eyes. Only 2 of the 23 flagged cases represented true consensus-GT errors, giving a residual GT error rate of 2/11, 488 ≈ 0.017% in the agree bucket.

This residual is two orders of magnitude smaller than the pipeline’s end-to-end field error rate measured below (∼1%), so the working GT is sharp enough not to dominate the pipeline-accuracy figures we report. It also has a more directly useful corollary: at the level of cross-field consistency, almost all violations on Pentacam printouts represent device-side measurement or computation artifacts rather than OCR errors; cross-field consistency is therefore not by itself a reliable indicator of OCR error, and the software does not use it to filter pipeline output.

As an additional, fully independent check, we drew a random sample of 60 agree-bucket fields (30 from the holdout and 30 from the Belin corpus, both generated by the PaddleOCR–Qwen procedure) and had two current-generation vision–language models independent of both OCR engines (Claude Sonnet and Opus) transcribe each field from its tight single-field crop. All 60 independent reads matched the accepted agree-bucket value, consistent with the residual-error estimate above and the DICOM SR cross-check below.

### Independent Verification Against DICOM SR

A subset of examinations carried DICOM SR objects providing the same measurements at machine precision (machine-precision floats in a coded sequence, independent of any rendering pipeline). For the 518 fields where both the SR and our pipeline returned comparable values, agreement was 100% within display rounding tolerance. Two additional cases were excluded as noncomparable: one in which a field (PupilDia) had value 0 in the SR but was not displayed on the printout (and was therefore correctly empty in OCR) and one in which a device-specific SR field mapping on a single device produced a value that differed from the displayed printout (and the OCR correctly read the printout). The 518-field match rate provides an OCR-independent confirmation of pipeline fidelity.

### Holdout Validation

The 200-examination holdout corpus, untouched during pipeline development, was processed end-to-end and evaluated against GT generated by the same Stage 2 procedure with human review of disagreements. Aggregate field-level accuracy on the holdout was 98.70%, within 0.76 percentage points of training accuracy (99.46%) across the three primary printout types. Accuracy concentration in residual errors and per-printout-type breakdowns are reported in Results.

## Results

### Pipeline Accuracy on the Three Validation Corpora

Field-level accuracy was assessed against the consensus-generated working ground truth (Methods) using exact-match comparison with a small per-field numerical tolerance. Results are summarized in Table 1 and visualized together with per-field reliability and cohort scale in Figure 3. On the 300-examination training corpus, the software achieved 99.46% field-level accuracy across all extracted fields. On the 200-examination internal holdout corpus, which played no role in pipeline development, accuracy was 98.70%—a degradation of 0.76 percentage points relative to training that is consistent with normal generalization rather than overfitting. On the 333-examination Belin/Ambrósio corpus—a printout type added to the framework only after the core pipeline had been frozen—accuracy was 99.03% (6124 of 6184 fields). Because an initial 135-examination batch was used to finalize the single Belin-specific crop parameter (Methods), we additionally report accuracy on the disjoint 198-examination batch that was held out until the extension was frozen: 99.29% (3655 of 3681 fields), marginally above the 135-examination development batch (98.64%). The Belin/Ambrósio extension thus provides a concrete demonstration that a new printout type can be added to the archetype-plus-affine framework—the most common extension we anticipate for users of the open-source tool—and reach production-grade accuracy on genuinely held-out data, without retraining or any change to the core OCR pipeline.

**Table 1.** Field-Level Accuracy by Validation Corpus and SR Cross-Verification

Corpus	Examinations	Fields Evaluated	Accuracy, %
Training (4 Maps/Topometric)	300	11,632	99.46
External holdout (4 Maps/Topometric)	200	7940	98.70
Belin/Ambrósio extension	333	6184	99.03
SR cross-verification	—	518 (paired)	100.00

**Figure 3.**
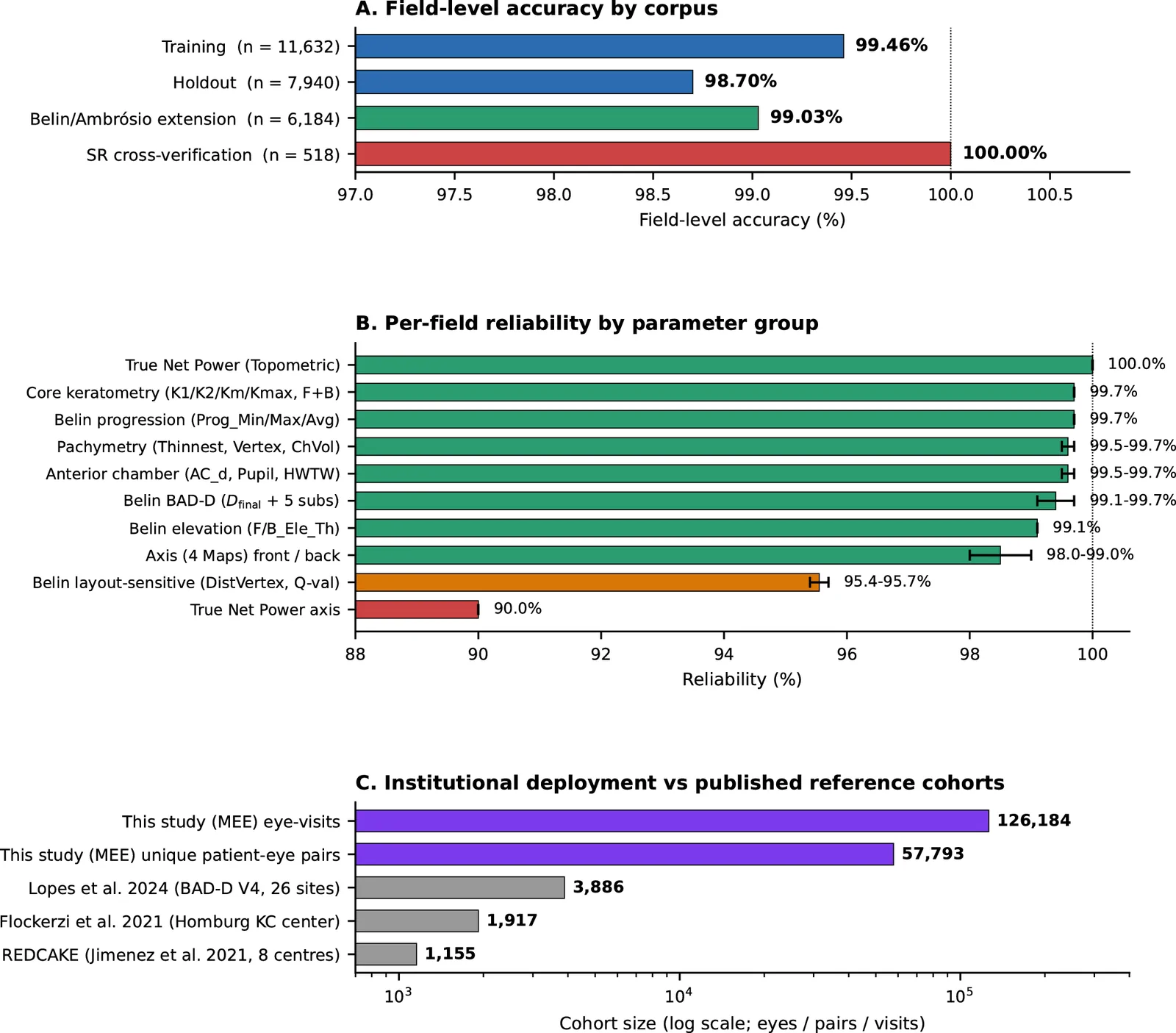
Validation summary for the *import_pentacam* pipeline. **(A)** Field-level extraction accuracy on the three validation corpora (training, internal holdout, Belin/Ambrósio extension) and on the independent DICOM SR cross-verification: 99.46%, 98.70%, 99.03%, and 100.00%, respectively. Holdout degradation relative to training is 0.76 percentage points, consistent with normal generalization; the SR cross-check is fully independent of any VLM-consensus or human-review machinery used to construct the working ground truth. **(B)** Per-field reliability by parameter group, calibrated on the validation corpora and embedded in the released software as a per-record signal. Bars span the observed reliability range across the corpora; the color-tier encoding (*green*, ⩾99%; *orange*, 95%–99%; *red* <95%) matches the REVIEW-flag thresholds used in the detailed output CSV. **(C)** Cohort scale comparison (log-scaled horizontal axis). The institutional deployment at one tertiary referral center (Massachusetts Eye and Ear) recovers measurements at a scale exceeding the three published multicenter reference cohorts by more than an order of magnitude. Eye visits = independent examination events; unique patient–eye pairs = laterality-resolved patient eyes contributing one or more visits.

For 518 field-level pairs where both an OCR reading and a DICOM SR value were independently available on the same examination, the two sources agreed in 100% of cases within display rounding tolerance. Two further paired cases were excluded as noncomparable: one in which a field (PupilDia) had value 0 in the SR but was not displayed on the printout (and was therefore correctly absent in OCR) and one in which a device-specific SR field-mapping anomaly on a single device produced a value that disagreed with the rendered printout (which the OCR correctly read). The SR cross-check is fully independent of any of the VLM-consensus or human-review machinery used to construct the working GT, and it provides instrument-level confirmation of the pipeline’s transcription fidelity.

### Per-Field Reliability

Per-field reliability scores were calibrated on the validation corpora and are embedded in the released software as a per-record signal in the detailed output CSV. Per-parameter accuracy—number available, correct, and incorrect—for the principal clinical parameters is given in Table 2; the exhaustive per-field breakdown for every extracted field is provided in Supplementary Tables S1 and S2.

**Table 2.** Per-Parameter Extraction Accuracy for the Principal Clinical Parameters, by Validation Corpus

Parameter	Corpus	*N*	Correct	Incorrect	Accuracy, %
Anterior K1 (D)	Training (300)	295	295	0	100.0
	Holdout (200)	200	200	0	100.0
Anterior K2 (D)	Training (300)	295	295	0	100.0
	Holdout (200)	200	200	0	100.0
*K*_max _ (D)	Training (300)	293	293	0	100.0
	Holdout (200)	200	200	0	100.0
Thinnest pachymetry/CCT (µm)	Training (300)	291	291	0	100.0
	Holdout (200)	199	199	0	100.0
Axis, anterior (°)	Training (300)	292	288	4	98.6
	Holdout (200)	197	179	18	90.9
Axis, posterior (°)	Training (300)	290	288	2	99.3
	Holdout (200)	197	176	21	89.3
BAD-D final score	Belin (333)	327	325	2	99.4
True Net Power power parameters^a^	Topometric	350	350	0	100.0
True Net Power axis (°)	Topometric	70	63	7	90.0

A Reading Is Counted Correct Only When it Matches the Value Printed on the Report to Displayed Precision; Extraction Is a Transcription Task, so Accuracy Is the Complete Performance Metric—a Deviation of any Size Is a Failure, not Measurement Noise. “Incorrect” Includes the Small Number of Fields the Pipeline Did not Read. The Exhaustive Per-Field Breakdown for Every Extracted Field Is Given in Supplementary Tables S1 and S2.

a The Five True Net Power Power Parameters Are K1, K2, K_m_, Astigmatism, and *P*_max_.

Core keratometry fields—K1, K2, K_mean_, and K_max_ on both anterior and posterior surfaces—reached 99.7% across all corpora. This is well within the published Pentacam HR measurement repeatability for these parameters^19^: pipeline-introduced uncertainty for keratometry is smaller than the instrument’s own measurement variability and is therefore clinically negligible for ectasia assessment. Pachymetry (Thinnest, PachyVertex), anterior chamber depth, and corneal-volume fields were similarly in the 99.5% to 99.7% range. The Belin/Ambrósio BAD-D composite score (*D*_final_) and the five subscores (*D*_*f*_, *D*_*b*_, *D*_*p*_, *D*_*t*_, *D*_*a*_) reached 99.1% to 99.7% across the Belin corpus.

Two Belin fields are lower-reliability: distance from corneal vertex to thinnest location (95.7%) and qualitative Q-value (95.4%). Both occupy compact regions of the Belin printout layout, where the target value is rendered immediately adjacent to other text; minor crop-placement variation propagates into occasional OCR misreads. The detailed output CSV flags fields with reliability below threshold as REVIEW so that downstream researchers can apply field-specific filtering.

Axis (front and back) is a methodologically interesting weak spot. The Pentacam printout renders Axis values in a hollow-stroked LCD-style display font on a colored background, the visually most ambiguous combination on the page. After OCR preprocessing (whitening, hollow-fill, 3× upscaling, archetype-positional crop rescue), Axis reaches the 98% to 99% range; the residual error is concentrated on rendered glyph configurations where the source font itself is genuinely ambiguous to the reader (e.g., 56° vs. 95°). This is an irreducible limit of the source rendering rather than of OCR alone: the same crops are misread at comparable rates by the larger frontier VLMs that contributed to ground-truth generation.

### Archetype Coverage in Production Deployment

The layout-normalization framework records, for every processed page, the residual standard deviation of the per-page affine fit between detected labels and the matched archetype (Methods). A high residual indicates a page whose layout is not consistent with any shipped archetype and is flagged for review. In the institutional deployment described below—comprising 255,016 input files and producing 421,423 raw extraction events—not a single examination produced an archetype-mismatch warning in the production log. Every encountered page fell within tolerance of one of the shipped archetypes (the two firmware-era 4 Maps/Topometric variants, the low-resolution PACS-export ∼904 px variant, the pre-2010 generation-1 layout, or the two Belin variants). This is empirical evidence that the small shipped archetype set covers the printout types and firmware variants actually present in a large clinical archive and that the framework’s residual-warning mechanism does not generate spurious flags at scale.

### Institutional Deployment: *import_pentacam* at Massachusetts Eye and Ear

As an application example, we deployed the software on the Pentacam archive of one tertiary corneal referral center (Massachusetts Eye and Ear, Boston, MA, USA). The deployment is intended to demonstrate the software’s applicability at institutional scale rather than to characterize the recovered dataset itself; comprehensive cohort characterization will be reported separately. The institutional PACS (Topcon Harmony, Topcon Healthcare, Inc., Oakland, NJ, USA) was queried for Pentacam examinations, returning a list of 255,016 archived files (a mix of DICOM objects, PDF, and image-format records). The software was run directly on the PACS file system, without copying any files to local storage and without any Harmony-specific configuration; while we have not tested deployment on other PACS products, no element of the input-file-handling pathway is Harmony-specific, and we expect the same approach to transfer. The software produced 421,423 raw extraction events—one row per (file, page, source)—which merged to **126,184 eye visit-level records covering 57,793 distinct patient–eye pairs from 29,550 unique patients** (Table 3). At least 10 distinct Pentacam devices were active across the institution’s satellite clinical sites, and the recovered archive spans 2008 through 2026 (18 years). Of the 421,423 raw extraction events, 93,565 read the SPR binary blob and 20,642 the DICOM Structured Report; a total of 114,207 raw extraction events therefore returned machine-precision values not subject to OCR uncertainty. The remaining 307,216 OCR-based extractions comprised 205,301 PDF pages embedded in DICOM objects and 101,915 standalone image-format PACS exports (JPG); the latter dominate the pre-2021 portion of the archive, reflecting the institution’s transition from image-based to DICOM-based archiving over the deployment span (Fig. 4A).

**Table 3.** Scale of the Institutional Deployment at Massachusetts Eye and Ear, Snapshot 2026-05-22

Quantity	Count
Input files ingested	255,016
Raw extraction events	421,423
From OCR of DICOM-embedded PDF pages	205,301
From OCR of standalone image files (JPG)	101,915
From the SPR binary blob	93,565
From the DICOM Structured Report	20,642
Eye visit-level records (compact output)	126,184
Unique patient–eye pairs	57,793
Unique patients	29,550
Pentacam devices encountered (institutional satellites)	⩾10
Archive years covered	2008–2026
Archetype-mismatch warnings	0

**Figure 4.**
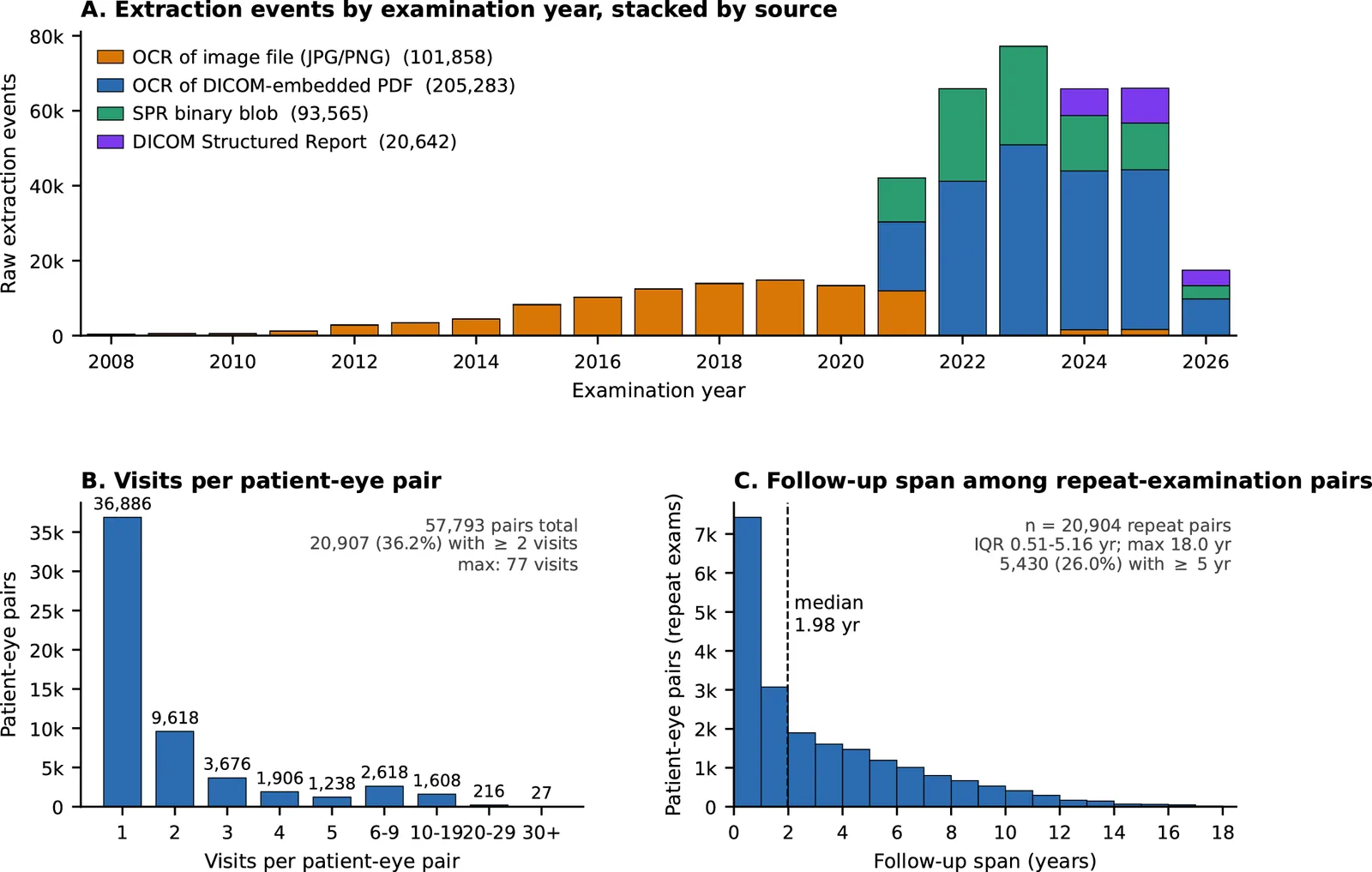
Composition and longitudinal structure of the institutional archive recovered by the deployment described in Table 3. **(A)** Raw extraction events by examination year, stacked by source: OCR of standalone image-format PACS exports (JPG/PNG; *orange*), OCR of the PDF page embedded in a DICOM object (*blue*), the SPR proprietary binary blob (*green*), and the DICOM Structured Report (*purple*). The archive transitioned from image-based to DICOM-based archiving in 2021: pre-2021 examinations are recovered almost exclusively by OCR of low-resolution image exports, whereas from 2022 onward, DICOM-embedded PDFs dominate, with machine-precision SPR data available on most examinations from 2021 and SR data on most examinations from 2024. The 2026 column is partial (examinations through May only). **(B)** Number of independent examination visits per patient–eye pair across the 57,793-pair cohort. Bin labels indicate the visit count or count range; 36.2% of pairs (20,907) have at least two visits, and individual pairs contribute up to 77 serial examinations. **(C)** Follow-up span (years between earliest and latest examination) among the 20,904 pairs with two or more dated visits. Median follow-up is 1.98 years (interquartile range 0.51–5.16 years); 26.0% of repeat-examination pairs have at least 5 years of recorded follow-up, with a maximum span of 18.0 years.

The deployment has been operated as a continuously running weekly job: examinations archived since the previous run are processed incrementally, and the cumulative output CSVs grow monotonically. The deployment has so far processed without any examination triggering an archetype-mismatch flag, layout warning, or OCR failure that the software could not recover from on retry.

For context, the largest published multicenter Pentacam ectasia validation cohort involved 3886 eyes from 26 international sites,^6^ the largest published single-center ABCD baseline cohort comprised 1917 corneas,^8^ and the REDCAKE multi center project enrolled 1155 eyes from 906 patients with keratoconus across eight contributing centers.^9^ The institutional deployment reported here, drawn entirely from routine clinical operation at one tertiary referral center, recovers measurements at a scale exceeding these published cohorts by more than an order of magnitude.

### Longitudinal Structure of the Recovered Archive

Of the 57,793 patient–eye pairs in the deployed output, 36.2% (20,907) had two or more independent examinations. Among the 20,904 of these repeat-examination pairs that carry two or more *dated* examinations, the median follow-up was 1.98 years (interquartile range 0.51–5.16; maximum 18.0 years), and 26.0% (5430) had at least 5 years of recorded follow-up. Individual patient–eye pairs contribute up to 77 serial examinations. This longitudinal depth was generated entirely by routine clinical practice over the 18-year span of the institutional archive, without prospective study enrollment. The full archive composition by year and extraction source, the distribution of visits per patient–eye pair, and the follow-up-span distribution among repeat-examination pairs are shown in Figure 4.

### Firmware-Era Stratification of Belin/Ambrósio Parameters

The Belin/Ambrósio Enhanced Ectasia Display indices entered the Pentacam software stack in successive firmware revisions over the 2010s. Examinations recorded on earlier device firmware therefore do not include a Belin/Ambrósio printout page and are intrinsically without Belin parameter values. In the institutional deployment, we observed *D*_final_ populated in 54.4% of eye visits from group A archetype (firmware ⩽1.28) examinations versus 96.3% from group B (firmware ⩾1.30) examinations—consistent with the firmware-era introduction of the indices and not with extraction failure (when the Belin page is present, Belin extraction reaches 99.03%; Table 1).

The archetype-group label that the software records in the detailed output CSV (Methods) is the natural stratification variable for retrospective analyses of the recovered dataset that use BAD-D scores or related Belin indices: Belin missingness in group A examinations should be interpreted as a structural property of the firmware era, not as a software failure mode.

## Software Summary

### Open-Source License

*import_pentacam* is distributed under the GNU Affero General Public License v3.0 or later (AGPL-3.0-or-later). The license applies to the complete source tree, the archetype definitions, the validation harness, and the worked examples provided in the repository.

### Generated Outputs

Each pipeline run produces three CSV files and one image directory per output location (Methods, *Output format*). pentacam_raw.csv records one row per extraction event (DICOM SR object, SPR binary blob, or OCR’d PDF or image page) and is written incrementally. pentacam_compact.csv records one row per eye-visit with the best available value per field after multisource merge, directly importable into statistical or analytics environments. pentacam_detailed.csv contains the same eye visit-level rows extended with per-field source, OCR confidence, reliability score, and QA flag. The companion images/ tree contains the four linked corneal-map PNG exports per eye-visit.

### Comparison With Other Open-Source Tools

To the best of our knowledge, no other open-source tool currently performs end-to-end extraction of Pentacam tomography measurements from clinical PACS records. Existing approaches are either commercial software bundled with the device (the Oculus Pentacam database export, which is operator-driven on a per-examination basis and requires interactive use of the manufacturer’s GUI) or device-specific extraction utilities described in the literature without a public source release. Open-source extraction tools for adjacent ophthalmic modalities exist—for example, for Humphrey visual-field reports^13^—but their scope is unrelated to Pentacam corneal tomography. A like-for-like baseline comparison against an existing open-source tool is therefore not currently possible; *import_pentacam* occupies a previously unfilled niche.

### External Usability Results

End-to-end accuracy was measured on three independent corpora totaling 833 examinations (Results, Table 1): 99.46% on the 300-examination training corpus, 98.70% on the 200-examination internal holdout, and 99.03% on the 333-examination Belin/Ambrósio extension corpus, with 100% agreement against 518 paired DICOM Structured Report fields within display-rounding tolerance. The institutional deployment at Massachusetts Eye and Ear (Table 3) has been operated as a continuously running weekly job, processing 421,423 raw extraction events from 255,016 input files without a single archetype-mismatch warning or unrecoverable OCR failure.

### Distribution

A Microsoft Windows MSI installer is provided for convenience (download link at https://github.com/tobiaselze/import_pentacam); for other operating systems, instructions for building from source are provided in the repository. *import_pentacam* is a self-contained end-user tool rather than a library intended for downstream Rust projects; submission to the crates.io Rust package registry is therefore not currently planned.

## Discussion

The release of *import_pentacam* addresses a structural data-access problem in clinical Pentacam research: the values that drive ectasia screening and refractive-surgery planning accumulate in institutional PACS as rasterized PDF printouts and proprietary binary readouts, not as structured DICOM tags or queryable database fields. We have shown that this gap can be closed automatically, at field-level accuracies of 98.70% to 99.46% on three independent corpora, on the scale of an entire institutional archive (here, 126,184 eye visits from a single tertiary center), and under continuous incremental operation.

The central methodological contribution is the archetype-plus-affine layout-normalization framework. The Pentacam printout layout is not a single fixed canvas: it has shifted across firmware releases, has accreted entirely new printout types over the past decade (Topometric/KC-Staging and Belin/Ambrósio), and has been re-rendered into low-resolution JPEG in some PACS configurations. A naive approach that hard-codes pixel locations is brittle across this history; label-matching alone loses any field whose label is mis-detected or rendered differently. By searching for labels first and falling back to per-page affine fits onto a small set of hand-defined archetypes, the software achieves three properties simultaneously: it handles existing layout variants without per-site configuration, as evidenced by the zero archetype-mismatch warnings in the 255,016-file institutional deployment; it remains extensible to layouts not seen at development time—the Belin/Ambrósio Ectasia Display was incorporated by adding one archetype and one label list after the rest of the pipeline was frozen and reached 99.03% accuracy on its own evaluation corpus; and it flags genuinely novel layouts via the affine-fit residual standard deviation, so that pages outside shipped archetype coverage are visible rather than silently misread.

A second methodological point is the convergence of independent validation lines. Pipeline accuracy on the 200-examination internal holdout (98.70%)—drawn from a different date range and PACS configuration than the training data—was within 0.76 percentage points of training-set accuracy, evidence that the framework generalizes across time periods and archiving configurations within the institution. The DICOM Structured Report cross-check, drawn entirely from outside any of the VLM- or human-review machinery used to build the working ground truth, agreed with the OCR output in 100% of 518 paired comparisons, providing an instrument-level confirmation of transcription fidelity. The cross-field consistency audit was instructive in an unexpected way: of 23 examinations flagged in the agree bucket, 21 turned out to reflect printout-level inconsistencies (failed measurements, computational overflow on edge-case eyes) that the software had correctly transcribed, and only 2 were true OCR errors. Cross-field consistency therefore does not serve as a reliable OCR-error detector; the software does not use it to filter values, and the audit’s principal value is in showing that the consensus-generated working ground truth is itself sharp to better than 1 part in 5000.

A third point concerns the operational character of the release. Published clinical-OCR pipelines have typically been one-shot batch extractions over fixed snapshots, including the closest precedent for this work—the automated extraction of Humphrey Visual Field report data,^13^ demonstrated in a single-printout-type, single-archive-format context. The software released here is structured as continuously operable research infrastructure: the institutional deployment has been running as an automated weekly update, with new examinations entering the structured output as they enter the PACS and with the cumulative output CSVs growing monotonically. This converts a one-off extraction artifact into an ongoing data resource, and it accommodates routine prospective follow-up of any subject already present in the archive without prospective recruitment.

A fourth point is multi-institutional deployability. The software is released under an open-source AGPL-3.0 license as a single statically linked Rust binary. It runs on Linux, macOS, and Windows without any external dependency installation; uses an NVIDIA GPU when one is available; and falls back transparently to the CPU otherwise. At our institution, it operates directly on the institutional PACS file system (Topcon Harmony) without any product-specific configuration. While we have not yet validated against other PACS products, no element of the input-file-handling pathway is Harmony-specific, and the file-content auto-detection (magic-byte format identification, fallback handling for nonstandard filename extensions) should transfer.

Finally, the recovered measurement archive at a single institution is itself a meaningful research resource. Eighteen years of routine clinical Pentacam imaging, recovered as a structured longitudinal dataset, sit comfortably outside the scale of the largest published single-center and multicenter Pentacam ectasia cohorts.^6^^,^^8^^,^^9^ It supports questions in keratoconus progression and refractive surgery outcome surveillance at a scale that prospective single-site recruitment cannot reach. Comprehensive characterization of that dataset—demographic structure, year-by-year measurement distribution, parameter availability by firmware era, and ectasia-indicator distributions at the patient level—is the subject of a separate analysis paper.

## Limitations

Several limitations of the software and of the recovered dataset should be noted.

First, the dataset described here as a deployment example originates from a single tertiary corneal referral center, whose clinical case mix is substantially enriched for corneal ectasia. The patient population comprises predominantly individuals referred for ectasia evaluation, refractive surgery candidacy, or complex contact-lens fitting, and is not a population sample. The proportion of examinations with elevated BAD-D, abnormal *K*_max_, or reduced pachymetry reflects this referral mix and not the prevalence of these findings in the general population.^1^^,^^2^ Parameter distributions from this dataset should not be used as normative reference values, and analyses based on it should be externally validated on less-enriched cohorts.

Second, the dataset contains no adjudicated diagnostic labels. Individual clinical diagnoses were not extracted in this retrospective work. The Belin/Ambrósio BAD-D and related ectasia indices are continuous topographic-deviation scores, not clinician-adjudicated diagnoses; research using datasets generated by this software for supervised classification should treat BAD-D–derived labels as surrogate outcomes and disclose this in any downstream report.

Third, residual extraction errors concentrate in a low-resolution PACS-export layout variant (approximately 1200 × 904 px). Fields with reliability below 96% (notably Belin Dist.Vertex and Q-value; Supplementary Table S2) are flagged REVIEW in the detailed output CSV so that researchers can apply field-specific filters at the analysis stage.

Fourth, several Pentacam printout types—the Holladay report, Cataract Pre-OP, Compare 2/4 Exams, Corneal Optical Densitometry, and the Belin/Ambrósio ABCD Progression Display—are detected by the printout-type classifier but are not currently extracted by the software. These are not extraction failures: the software correctly identifies the page type and skips them. They represent unsupported printout coverage that future archetype additions could close.

Fifth, Pentacam firmware updates have recalibrated the BAD-D normative reference across the 1.28/1.30 firmware boundary, and analyses spanning that boundary should stratify by the archetype-group label provided in the detailed output CSV (Methods).

Sixth, the software has so far been validated against DICOM, PDF, and image exports from a single PACS product (Topcon Harmony). While the input-handling pathway is not Harmony-specific—files are identified by magic bytes rather than by vendor conventions, and the embedded PDF and private DICOM tags parsed by the software are written by the Pentacam device rather than by the PACS—we have not verified extraction against raw DICOM exports from other PACS vendors. Multivendor PACS compatibility is therefore a target for future multi center testing, and we would welcome such validation as part of external adoption of the tool.

Seventh, our per-field ground truth was established primarily by agreement between two automated readers (PaddleOCR and Qwen 2.5-VL) with human review of disagreements. Because PaddleOCR is also the OCR engine used by the released pipeline, this ground truth is not fully independent of the system being evaluated, and agreement in the “agree” bucket could in principle mask a shared error. Two considerations bound this risk. The two engines are architecturally unrelated—a convolutional text detector-plus-recognizer versus a vision–language transformer—and fail in largely uncorrelated ways, so shared errors are rare. More importantly, the DICOM Structured Report cross-check is completely independent of both OCR engines and agreed with the pipeline on 100% of 518 machine-precision fields, while the cross-field consistency audit identified only two true errors among the 11,488 agree-bucket fields (0.017 %). We nonetheless state this dependence explicitly. Two further points bound it: for the majority of the training corpus (the testset3 and new_testset sub-corpora, 180 of 295 examinations), the reference ground truth was itself established by the independent Sonnet+Opus dual-VLM consensus rather than by PaddleOCR (Methods), and we verified a random sample of 60 agree-bucket fields (30 holdout, 30 Belin) with two vision–language models independent of both OCR engines, all 60 of which matched the accepted value (Validation).

Finally, comprehensive characterization of the recovered dataset—longitudinal follow-up distribution, demographic structure, parameter missingness by year and firmware era, and descriptive cohort statistics—is ongoing and will be reported in a separate analysis paper.

## Conclusions

We present *import_pentacam*, an open-source, platform-independent Rust tool that recovers structured corneal tomography measurements from clinical Pentacam DICOM, PDF, and image-format records. Field-level accuracy is 98.70% to 99.46% across three independent validation corpora, agreement with the machine-precision DICOM Structured Report is 100% on 518 paired comparisons, and the framework extends to printout types added after pipeline freeze without retraining. Deployment at one tertiary referral center over an 18-year archive has recovered measurements from 126,184 eye visits and 29,550 patients—a scale exceeding the largest published single-center Pentacam cohorts by more than an order of magnitude—and is operated as a continuously running weekly update. The software is openly released and is deployable at other Pentacam-equipped institutions using standard PACS infrastructure, without prospective recruitment.

## Software Repository

The software is hosted at https://github.com/tobiaselze/import_pentacam under the GNU Affero General Public License v3.0 or later (AGPL-3.0-or-later). The repository contains:

• the complete Rust source tree and build configuration (Cargo.toml), with the archetype definitions and per-page affine-fit logic exposed as inspectable source rather than as opaque trained weights;
• a Microsoft Windows MSI installer for convenience (download link in the repository README), with instructions for building from source on other operating systems;
• installation documentation (INSTALL, with prerequisites and build instructions) and built-in command-line help (import_pentacam –help) covering the supported invocation modes, the supported printout types, and the three output CSV files; and
• the validation harness, ground-truth annotation specifications, and consensus-generation logic used to produce the accuracy and per-field reliability numbers reported in Results.

###  

#### Usage Examples

The four supported invocation modes are illustrated by the following examples:

**Table:** 

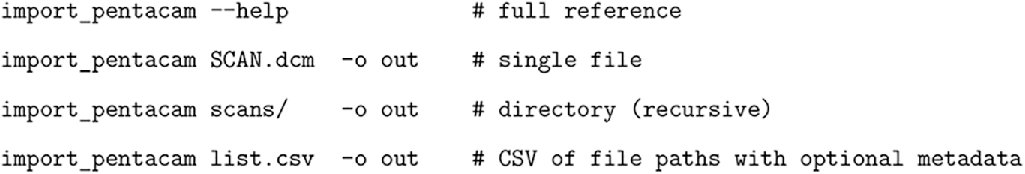

A DOI link to the published article will be added to the repository once the article is accepted.

## Supplementary Material

Supplement 1
