# Supplementary material for: import_pentacam: Open-Source OCR Recovery of Corneal Tomography Measurements From Pentacam DICOM, PDF, and Image Archives
Source: Transl Vis Sci Technol. 2026 Oct 27;15(10):46. doi: 10.1167/tvst.15.10.46 (PMC13730113; doi:10.1167/tvst.15.10.46)
Supplement: Supplementary file 1 [file tvst-15-10-46_s001.pdf]

Supplementary Material

import_pentacam: Open-Source OCR Recovery of Corneal Tomography Measurements from Pentacam DICOM, PDF, and Image Archives

Supplementary Tables S1 and S2 give the complete per-field extraction accuracy of the released (Rust) pipeline for every extracted field, complementing the principal-parameter summary in Table 2 of the main text. A reading is counted correct only when it matches the value printed on the report to displayed precision (absolute tolerance 0.05 in field units; 0.5 for integer-display micrometer fields). "Incorrect" includes fields the pipeline did not read (reported separately as "No-read" for the Belin corpus in Table S2). Because extraction is a transcription task, a deviation of any magnitude is counted as a failure; error magnitude is therefore not a meaningful per-field quantity and is not reported.

Table S1: Per-field extraction accuracy on the training and holdout corpora (4 Maps / Topometric fields), Rust pipeline.

	Training (300)				Holdout (200)			
**Field**	N	Corr.	Inc.	Acc.%	N	Corr.	Inc.	Acc.%
Rf_front	295	292	3	99.0	199	195	4	98.0
Rs_front	295	294	1	99.7	200	192	8	96.0
Rm_front	293	286	7	97.6	200	190	10	95.0
K1_front	295	295	0	100.0	200	200	0	100.0
K2_front	295	295	0	100.0	200	200	0	100.0
Km_front	295	295	0	100.0	200	200	0	100.0
Astig_front	295	289	6	98.0	200	198	2	99.0
Axis_front	292	288	4	98.6	197	179	18	90.9
Qval_front	293	293	0	100.0	200	197	3	98.5
Rmin_front	295	295	0	100.0	200	200	0	100.0
Rper_front	293	293	0	100.0	200	200	0	100.0
Rf_back	288	286	2	99.3	200	192	8	96.0
Rs_back	292	292	0	100.0	199	195	4	98.0
Rm_back	293	286	7	97.6	200	196	4	98.0
K1_back	295	293	2	99.3	200	199	1	99.5
K2_back	295	293	2	99.3	200	199	1	99.5
Km_back	295	292	3	99.0	200	198	2	99.0
Astig_back	295	293	2	99.3	200	199	1	99.5
Axis_back	290	288	2	99.3	197	176	21	89.3
Qval_back	293	293	0	100.0	200	199	1	99.5
Rmin_back	293	293	0	100.0	200	200	0	100.0
Rper_back	295	293	2	99.3	200	200	0	100.0
PupilCenter	289	288	1	99.7	197	196	1	99.5
PupilCenter_x	291	290	1	99.7	198	195	3	98.5
PupilCenter_y	294	292	2	99.3	198	198	0	100.0
PachyVertex	290	288	2	99.3	197	197	0	100.0
PachyVertex_x	293	290	3	99.0	200	198	2	99.0
PachyVertex_y	295	295	0	100.0	200	200	0	100.0
Thinnest	291	291	0	100.0	199	199	0	100.0
Thinnest_x	293	292	1	99.7	200	199	1	99.5
Thinnest_y	295	294	1	99.7	200	199	1	99.5
Kmax	293	293	0	100.0	200	200	0	100.0
Kmax_x	293	292	1	99.7	200	200	0	100.0
Kmax_y	295	289	6	98.0	200	199	1	99.5
CorneaVol	293	293	0	100.0	200	200	0	100.0
HWTW	246	244	2	99.2	167	163	4	97.6
ChamberVol	293	293	0	100.0	200	200	0	100.0
Angle	291	291	0	100.0	199	198	1	99.5
AC_depth	282	282	0	100.0	195	194	1	99.5
PupilDia	292	292	0	100.0	198	198	0	100.0

Table S2: Per-field extraction accuracy on the Belin/Ambrósio corpus (333 examinations), Rust pipeline. "No-read" counts fields the pipeline did not return a value for; these are included in "Incorrect".

**Field**	N	Correct	Incorrect	No-read	Acc.%
K1	332	331	1	1	99.7
K2	332	331	1	0	99.7
KMax	332	331	1	0	99.7
Axis	333	329	4	0	98.8
Qval	329	314	15	3	95.4
PachyThin	331	329	2	1	99.4
DistVertex	280	268	12	0	95.7
F_Ele_Th	327	324	3	1	99.1
B_Ele_Th	327	322	5	2	98.5
Prog_Min	332	331	1	0	99.7
Prog_Max	330	329	1	0	99.7
Prog_Avg	332	331	1	0	99.7
ARTmax	332	330	2	0	99.4
Df	323	321	2	0	99.4
Db	325	324	1	0	99.7
Dp	325	322	3	0	99.1
Dt	310	309	1	0	99.7
Da	325	323	2	1	99.4
D_final	327	325	2	0	99.4
